# Straw as an Alternative to Grass Forage in Horses—Effects on Post-Prandial Metabolic Profile, Energy Intake, Behaviour and Gastric Ulceration

**DOI:** 10.3390/ani11082197

**Published:** 2021-07-24

**Authors:** Anna Jansson, Patricia Harris, Sara Larsdotter Davey, Nanna Luthersson, Sveinn Ragnarsson, Sara Ringmark

**Affiliations:** 1 Department of Anatomy, Physiology and Biochemistry, Swedish University of Agricultural Sciences, 75007 Uppsala, Sweden; anna.jansson@slu.se; 2 WALTHAM Petcare Science Institute Waltham-on-the Wolds, Melton Mowbray, Leics LE14 4RT, UK; pat.harris@effem.com; 3 University Animal Hospital, Swedish University of Agricultural Sciences, 75007 Uppsala, Sweden; sara.larsdotter.davey@slu.se; 4 Hestedoktoren I/S, Hvalsøvej 298, 4360 Kr. Eskilstrup, Denmark; nanna@hestedoktoren.dk; 5 Department of Equine Science, Hólar University, IS-551 Sauðárkrókur, Iceland; sveinn@holar.is

**Keywords:** gastric ulcer, insulin, satiety, straw, serotonin, weight loss

## Abstract

**Simple Summary:**

Many leisure horses have low energy requirements and obesity is common. Straw has a low energy content and could be a forage option for these horses. However, a previous study suggested that providing straw as the only forage was associated with an increased risk for gastric ulcers. This study evaluated replacing 50% of the daily forage allowance with a good hygienic quality wheat straw. Six horses were fed both the control diet (grass forage only, CON) and the straw diet (50:50 grass forage and straw, S). Each diet was fed for three weeks and all horses were evaluated on both diets. Diet did not affect the prevalence of gastric ulcers. Feed intake time was longer and daily energy intake lower on diet S, compared to CON. Plasma insulin levels were lower on diet S compared to CON, which could be beneficial for horses with overweight or insulin dysregulation. The results suggest that good hygienic wheat straw provided at 50% of the forage ration does not cause gastric ulcers, but may prolong feeding time and promote a metabolic profile more suitable for overweight horses. Including straw as part of the ration therefore may improve welfare for horses with low energy requirements.

**Abstract:**

Straw’s low energy content means it is a roughage option for horses with low energy requirements. Previously, in a field study, straw was associated with an increased risk for gastric ulcers. This study evaluated the effect on gastric ulcers, metabolic profile and behaviour of replacing, in a forage-only ration, 50% of the daily allowance with wheat straw. Six equines were studied in a 2 × 21-day cross-over design. The control diet (CON: 100% grass forage) and the straw diet (S: 50% grass forage and 50% straw [DM basis]) were iso-energetic. Gastroscopy was performed prior to the study and on day 21 and blood samples were collected and behavioural observations were performed. Diet did not affect squamous or glandular gastric ulcer scores (*p* > 0.05). Feed intake time was longer (*p* < 0.05) plus energy intake and plasma insulin concentrations were lower on diet S compared to CON (*p* < 0.0001). Plasma serotonin concentrations tended to be higher on diet S compared to CON (*p* = 0.05). The results suggest that good hygienic quality wheat straw can be included for up to 50% of the diet without causing gastric ulcers and that it can extend feeding time and promote a metabolic profile more suitable for overweight horses.

## 1. Introduction

The use of straw as a feedstuff for horses and ruminants has a long history. In 1949, Olsson et al. [1] reviewed the literature and described the nutrient composition and digestibility coefficients of oat, wheat, and rye straw from 49 studies performed on horses in different countries. The paper concluded that in contrast to most other feeds used, straw generally contained little digestible protein, had a comparatively high fibre content, low organic matter digestibility and accordingly, a low digestible energy content. The use of straw treated with ammonia to improve organic matter and protein digestibility has been studied [2] and untreated, as well as ammoniated wheat straw has been reported acceptable to horses at a level of 50% of a forage diet [3].

Historically, the use of straw in the diets of horses and ruminants has been a means of providing the necessary roughage for the animals and to make use of an agricultural by-product. However, today, the use of straw in diets for horses may be a means of improving animal welfare. Many leisure horses have low energy requirements and obesity is a common condition [4,5]. Dietary energy restriction is often necessary and that compromises natural feed intake patterns [6]. The intake of straw is slower than the intake of hay and cereals [7] and therefore total daily eating time is expected to increase when straw is included in a diet. Several studies indicate that the presence of stereotypic behaviours is associated with diets low in roughage [8,9]. In a survey by Hockenhull and Creighton [10] behaviours associated with frustration and aggression were common in connection with feeding, but the survey also indicated that these behaviours were less common in horses offered comparatively large amounts of roughage. It was recently suggested that horses should be fed at least 15 g dry matter (DM) of forage per kg of body weight (BW) and day in order to support natural behaviour and health [6]. However, for some horses, even this amount of grass or legume forage may exceed energy requirements especially during a weight loss programme and therefore replacing part of the forage ration with low energy straw could be a good alternative. However, in a field study on privately owned horses, the use of straw as the only roughage was associated with increased risk of gastric ulcers [11] and the authors suggested that mechanical damage, changes in the gastric content “mat” and/or a reduction in buffering capacity due to the low calcium and protein content of straw could have been the cause. Alternative explanations are also possible, including hygienic quality. Straw is known to be at risk of low hygienic quality, i.e., contain fungi, mesophilic bacteria and actinomycetes which are associated with health problems [6,12,13]. Straw batches collected from horse stables may contain significant amounts of microbes, mycotoxins (e.g., deoxynivalenol) and lipopolysaccharides [13]. Little is known about how such factors affect the long term gastrointestinal health in horses. Further studies, therefore, are needed to confirm any causality between straw feeding and gastric ulcers. The aim of this study was to evaluate how replacing half the daily grass forage allowance with straw would affect gastric ulceration, feeding behaviour and post prandial metabolites. The hypothesis was that replacing 50% of a forage only ration with good hygienic quality straw would not increase the score for severity and number of gastric ulcer lesions but would alter the metabolic response and increase the time spent foraging.

## 2. Materials and Methods

The study was conducted in November−January 2018–2019 in Uppsala, Sweden at the facilities of the Swedish University of Agricultural Science. Ethical permission was given by the Uppsala ethical committee dnr: 5.8.18-14033/2018.

### 2.1. Horses

Six adult animals (3–15 y, 220–570 kg of body weight (BW), body condition score (BCS) 5–8/9 [14] with no owner reporting current clinical issues and no clinical history of colic or gastric ulceration were used for the study. All animals were used to being fed haylage but not straw and all had been out of training for at least 6 months prior to the study. They came from three different yards (two from each yard) and arrived at the study location five days before the initial gastroscopy. Four had received an anthelmintic treatment four weeks before they arrived at the study site and the other two received anthelmintic treatment three days prior to the initial gastroscopy.

### 2.2. Study Design and Diets

**Figure 1:**
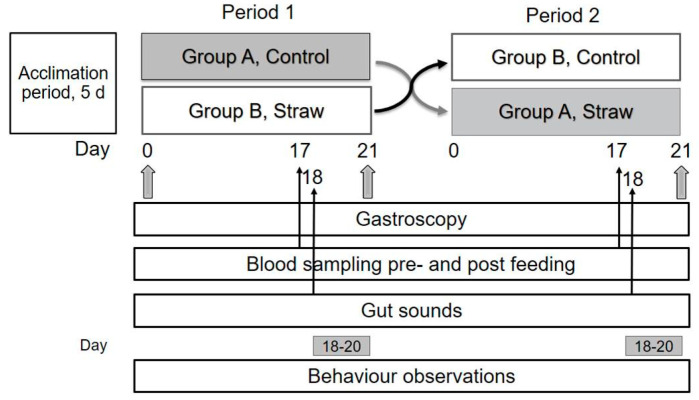
Illustration of the cross-over study design (three horses in group A and B, respectively, that shifted diets from period 1 to 2). Days for data collection in each period are illustrated by the numbers and arrows. Periods were following each other with no days in between. The gastroscopy performed on Day 0 was used to make group A and B as equal as possible with respect to initial ulcers scores.

The study was performed as a 2 × 21-day cross-over study with an initial adaptation period of five days (Figure 1). The length of the periods was based on observations that gastric ulcers may be affected by diet after 14 days of feeding [15]. After the acclimation period, the gastric mucosa was examined by gastroscopy and squamous lesions were graded 0–4 according to Sykes et al. [16]. Glandular lesions were graded from 0–2 according to Sykes et al. [17]. Horses were then divided into two groups balanced for the number and severity of gastric lesions and stable of origin. This resulted in a median score and interquartile range (IQR) for squamous gastric lesions of 2.5 IQR 1 in group A and 2.5 IQR 1.75 in group B and a median score and IQR for glandular gastric lesions of 1 IQR 0.75 in group A and 1 IQR 0.75 in group B (see Figure 1 for explanation).

Three feeds were used to formulate the study diets (Table 1), an early cut immature haylage (H1, mainly timothy and meadow fescue), a late cut more mature haylage (H2, mainly Italian Ryegrass (50% tetraploid and 50% diploid)) and wheat straw. The haylages were chosen to balance the low digestible protein content of straw (H1) and to make the diets as similar as possible with respect to energy and DM intake (H2). All feeds were analysed prior to the study with respect to hygienic quality (Eurofins Agro Testing Sweden, Kristianstad, Sweden) without any adverse remarks regarding content of mould, yeast or bacteria according to the laboratory guidelines (Straw (log colony forming units (cfu)/g): aerobic microorganisms 7, moulds < 5, Aspergillus fumigatus < 1; haylages (log cfu/g): Escherichia coli < 1, Enterobacteriaceae < 2, yeast 5, moulds < 2–3, Aspergillus fumigatus < 1, spores from butyric acid formers < 3, aerobic spore formers < 3–4, pH 4.9). To determine DM content and intake, feed samples and samples of feed refusals were dried (60 °C for 24 h and 103 °C for 16 h). Analysis of nutritional composition of forage samples was performed by NIRS (FOSS, Hilleröd, Sweden) that 207 was calibrated for airtight stored forage with DM contents 208 of 35% to 65% and hay with DM contents of 75% to 85%. at Agrilab AB, Uppsala, Sweden. Macronutrient concentrations were analyzed by inductively coupled plasma-atomic emission spectroscopy (ICP-AES) using Spectro Flame equipment (SPECTRO Analytical Instruments, Kleve, Germany).

During the five-day acclimation period, horses were fed all three types of feed (50% H1, 25% H2 and 25% straw) to facilitate a smooth introduction of the study diet as soon as the first study period started.

For the study itself the control diet (CON) consisted of 50% H1 and 50% H2 on a dry matter (DM) basis and was fed at 1.22 ± 0.01 kg per 100 kg BW. The straw diet (S) consisted of 49% H1, 1% H2 and 50% straw on a DM basis and was fed at 1.43 ± 0.01 kg per 100 kg BW. The change between the study diets was made abruptly (no days of adaptation) and 1% of H2 was included in the straw diet to ease the introduction of this haylage when switched to the control diet. Diets were complemented with 17 g/100 kg BW of a commercial mineral and vitamin (A, D and E) balancer (Krafft Miner Balancer, Krafft AB, Falkenberg, Sweden) and 5 g of NaCl/100 kg body weight (BW). Diets were balanced with respect to metabolisable energy (ME) intake (±2%, *p* > 0.05) and met the horse’s estimated requirements of ME but minimise potential weight gain [18], crude protein (CP), Ca, P, Mg, Se and NaCl according to NRC [19] (Table 2). The energy allowance was calculated based on metabolic weight and the allowance adjusted to fulfil a minimum of 1% dry matter of BW. The final feed allowance was rounded to the nearest 10 g.

### 2.3. Management

Horses were kept in single box stalls with rubber mats and sand on the floor between 15.00 and 09.00. They spent the rest of the time (09.00–15.00) outside in sand paddocks (180 m^2^) in pairs with the horse from the same yard. Water was provided ad libitum from buckets both inside and in the paddocks but no feed or forage were available while in the paddocks.

The forage was provided at 07.00, 15.00 and 22.00 from hay nets (3.5 × 3.5 cm openings) to five of the horses to minimise feed spoilage and on the floor to one of the horses with a slow consumption rate. Three horses were also fed all or part of their straw ration on the floor to facilitate consumption. The feed was distributed so that ~50% of H1 was fed at 07.00 and 25% of H1 was fed at 15.00 and 22.00, respectively. The straw and H2 were distributed evenly between the 15.00 and 22.00 feedings. Leftovers were collected daily and weighed on a weekly basis for each individual.

Body weight (BW) was monitored using a weighbridge once per week. In week 1 and week 3 of each period, body condition was assessed by the same trained assessor according to Henneke et al. [14] but divided into sections as earlier described by Ringmark et al. [20].

### 2.4. Examination of Gastric Mucosa

Gastroscopy was performed by an experienced clinician (observer 1) on three occasions, before period 1 and at the end of both period 1 and 2, using a 3.25 m flexible video endoscope (PV-G 34-325, Karl Storz SE & Co. KG, Tuttligen, Germany). Examinations were video recorded and sent to an external observer (observer 2) and both the examining clinician and the external observer were asked to grade and describe any lesions in the squamous mucosa according to the Equine Gastric Ulcer Council 0–4 scoring system (ESGD, [16]). Lesions in the glandular mucosa (EGGD) were graded from 0–2 according to Sykes et al. [17]. Both observers were blinded to which diet horses were fed at the time of examination and the external observer was also blinded to the order of the examinations.

### 2.5. Blood Sampling and Plasma Analyses

On day 17 in each period, a permanent catheter was placed in the jugular vein under local anaesthesia at 13.00 and blood samples were then collected into lithium heparin (5 mL) and EDTA (5 mL), before both the 15.00 and 22.00 feeding occasions and at 90 and 120 min after each feeding occasion. Samples were kept on ice and centrifuged for 15 min at 2700 rpm, 920× *g* within 30 min. Plasma was collected, and the total plasma protein concentration (TPP) determined using a refractometer (Atago, Tokyo, Japan) before storage at −20 °C until further analyses.

For determination of plasma serotonin and cortisol concentration, competitive ELISA assays were used (Serotonin ELISA and cortisol ELISA, IBL International (Tecan AB, Stockholm, Sweden) with intra-assay CV 3.7% and 8.2%, respectively. Plasma glucose concentrations were analysed with an enzymatic colorimetric/UV-method (D-Glucose, UV-method, Boeringer Mannheim, Germany, intra-assay CV 10.0%). Plasma insulin was analysed using a sandwich ELISA method (Mercodia Equine Insulin ELISA, Animal insulin control, Mercodica, Sweden, intra-assay CV 6.5%). Plasma non-esterified fatty acids (NEFA) were analysed by an enzymatic colorimetric assay (NEFA-HR(2) R1 set, NEFA-HR(2) R2 set, NEFA standard, Waco Control serum1, Fujifilm Wako Chemical Europe GmbH, Germany) which has been successfully used in earlier cross-over studies on horses [21,22]. In this study the intra-assay CV was 11.7%. Plasma acetate was analysed by a colorimetric assay for microtiter plates (Acetate Colorimetric Assay Kit, Sigma-Aldrich, Germany, intra-assay CV 10.8%). Occasionally, the CV for duplicates was high and only samples with CV < 23% were included in the analysis. Plasma urea was analysed by a colorimetric assay for microtiter plates (Urea Assay Kit, Cell Biolabs Inc, San Diego, CA, USA, intra-assay CV 12.3%).

### 2.6. Intestinal Sounds and Faecal Sampling

The animal care takers were instructed to pay special attention to abnormal behaviours and defecation patterns throughout the study. In order to provide an indicator of intestinal activity, intestinal sounds were recorded behind the last rib in both the upper and the lower quadrant and in both the right and the left side over 60 s in each location on day 18 of each period by the same person using a stethoscope. All sounds with a distinct beginning and end were counted. In addition, faecal dry matter content was analysed in a sample from each horse collected from the floor immediately after defecation on day 19 of each period. The samples were stored at −20 °C in plastic bags and dry matter content was subsequently determined by drying the samples at 60 °C for 24h.

### 2.7. Feeding Time, Prefeeding Behaviour and Heart Rate

During the first and last week of each period, the time spent ingesting as well as the rate of feed consumption was recorded for all animals with respect to the 15.00 feeding occasion. Horses with BW 450–550 kg were fed 1 kg of DM and horses with BW 200–250 kg were fed 0.5 kg DM consisting of either 50% H1 and 50% H2 (horses on diet CON) or 50% H1 and 50% straw (horses on diet S). Feed DM content was established before each feeding by taking a small forage sample and weighing it fresh out of the bale to be used. The sample was then heated in a microwave until all the water had evaporated and the weight of the sample stayed at a constant. The time required for total consumption as well as feeding pauses, i.e., when horses were not chewing or nibbling at feed, was noted. After 1h any remaining feed leftovers were collected and weighed.

Prefeeding behaviour was registered for all animals with respect to the 22.00 feeding. Two horses per day were observed during day 18–20 in each period. The observer entered the stable and sat quietly in a position where the two horses could be easily observed. After 15 min, when the horses no longer appeared to have any interest in the observer, all behaviours undertaken during the following 15 min were noted. Behaviours related to the mouth but not involving feeding (yawning, licking lips and sniffing floor) were included in a category of “oral behaviours”. The “motion behaviours” included walking around, pawing and scratching.

For the same two horses that were subjected to the behavioural observations on that day, a heart rate (HR) recorder was applied from around 16.00 until 09.00 the next morning using Polar M460 and Polar H7 heart rate girth (Polar Electro Oy, Kempele, Finland). The data were processed by Polar flow (https://flow.polar.com, accessed on 1 February 2019) and exported to Microsoft Excel, Windows 10 ver 1909, Microsoft Corporation, Redmond, WA, USA). From this, mean HR 30 min prior to the 22.00 feeding was calculated, (i.e., around five h after the HR-recorders were applied) as a measure of activity and/or stress level as it has earlier been observed that different feeding strategies may cause changes including aggression [23].

### 2.8. Statistical Analyses

All statistical analyses were performed in SAS 9.4 (SAS, Cary, NC, USA) and the level of statistical significance was set at *p* < 0.05 (presented as <0.05, <0.01, <0.001 and <0.0001) with statistical tendencies (>0.05, *p* < 0.1) reported with exact *p*-values.

The nutrient composition of the diets as fed per 100 kg BW and day were analysed for differences between treatments using analysis of variance and a general linear model including the effect of diet. The gastric ulcer scores followed multinominal distribution and therefore a generalised mixed model with a multinominal distribution and a cumulative probit link, i.e., a so-called proportional odds model, was used including the fixed effects of diet, period and assessor. The change in score between diets was analysed with a model including the effects of diet and assessor. One assessment was lacking from one of the assessors in period 2 due to bad video quality. Results are presented as the number of observations in each score.

For the parameters BW, BCS, nutrient intake and feeding time, where there were more than one observation per horse and period, a mixed model, with the effect of diet, period, and interaction of period*diet where a horse was treated as repeated measurements and a random effect, was used with an autoregressive covariance structure. For analysis of feeding time and feed and nutrient intake, the effect of week (1, 2 or 3) within period was also included in the model as repeated measurements, as it was expected that horses might need some time to get used to the new diet before consuming the whole ration. The same model, but with a univariate covariance structure, were used for the parameters calculated difference in BW and BCS within each period, intestinal sounds, faecal dry matter, heart rate and prefeeding behaviour.

Blood parameters were analysed using a mixed model including effects of diet, period, sample and an interaction of feeding, sample and diet. Horses were treated as a random effect and horse, period and sample were set as repeated measurements following an autoregressive covariance structure. Additionally, feeding occasions (15.00 or 22.00) were also analysed separately for effect of horse, period, sample and diet including an effect of interaction between sample and diet. One of the horses showed evidence of insulin dysregulation [24] with basal plasma insulin levels four times higher than the mean of the others. It was therefore excluded from the statistical analysis of plasma insulin and plasma glucose concentration. This individual had a history of previous laminitis although it had not had an episode within 6 months of the study. Results are presented as least square means ± SE.

## 3. Results

All animals fulfilled the study and remained healthy throughout the study period and no abnormal behaviours or changes in defaecation patterns were noted by the caregivers.

### 3.1. Nutrient Intake and Body Condition

Mean intakes per 100 kg BW of DM, ME, digestible CP, CP and NDF for both diets are summarised in Table 3. As some horses did not consume the whole ration, intake of ME, CP, WSC and dCP were lower when fed diet S than when fed diet CON. No difference was detected in feed intake between the weeks within a period nor any effect of interaction between week and diet. There was no difference in BW between the two diets (C: 323 ± 67 kg, S: 320 ± 67 kg, *p* < 0.001) nor in the change in BW (C: −0.6 ± 2.0 kg, S: −2.3 ± 2.0 kg, *p* > 0.05). Neither BCS (C: 5.5 ± 0.5, S: 5.4 ± 0.5, *p* > 0.05) nor change in BCS were affected by the diet (C: +0.1 ± 0.1, S: −0.2 ± 0.1, *p* > 0.05).

### 3.2. Gastric Ulcers

**Figure 2:**
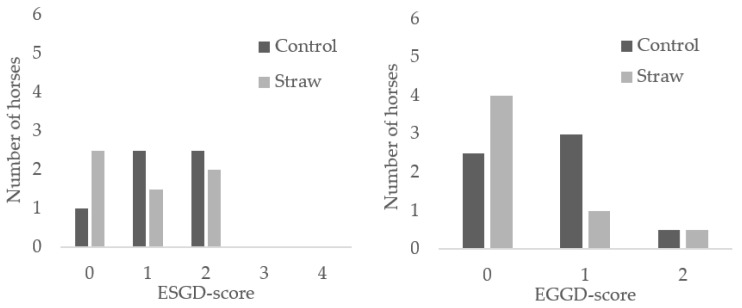
Number of observations (horses) in each score for equine squamous gastric disease (ESGD) and glandular equine glandular gastric disease (EGGD) in six horses fed a control diet (grass haylage only) and a diet with 50% wheat straw and 50% grass haylage (of DM) for three weeks each in a 2 × 21-day cross-over design.

There was no difference between assessors and therefore results were combined and presented as the mean. Diet did not affect the prevalence of either squamous, i.e., ESGD, or glandular, i.e., EGGD gastric ulcers (*p* > 0.05, Figure 2), nor was there a change in the score from the previous diet (ESGD; change during S: −1.2 ± 0.9, change during CON: −0.7 ± 1.0, EGGD; change during S: −0.3 ± 0.6; change during CON: −0.2 ± 0.8, *p* > 0.05). There was an effect of period on ESGD but not on EGGD. ESGD score decreased (*p* < 0.05) between period 1 and period 2 (1.3 ± 0.8 vs. 0.8 ± 0.9, (mean ± std)) while EGGD score remained unchanged (*p* > 0.05).

### 3.3. Intestinal Sounds and Faecal Dry Matter

Faecal dry matter content was not affected by diet (CON: 18 ± 1% and S: 20 ± 1%, *p* > 0.05). No difference was detected in the number of intestinal sounds between the diets (CON: 32 ± 3 and S: 33 ± 3 sounds/min, *p* > 0.05).

### 3.4. Plasma Insulin, TPP and Metabolite Concentration

**Figure 3:**
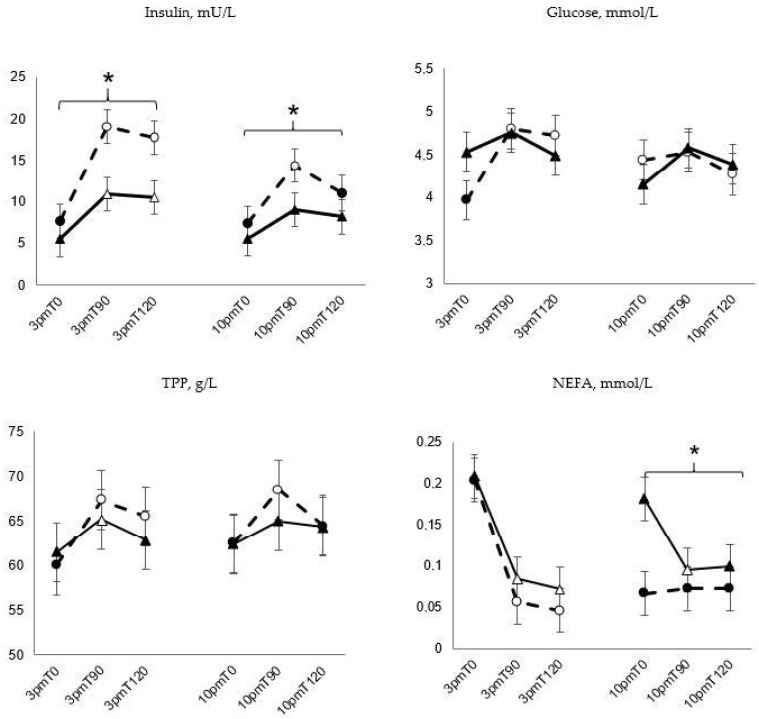
Plasma concentrations of insulin, glucose, NEFA and total plasma proteins (TPP) at time (T) 0, 90 and 120 min after feeding (LSmeans ± SE) in six horses (five horses included in the analysis of plasma insulin and glucose) subjected to a control (grass haylage only) diet (●) and a diet with 50% wheat straw and 50% grass haylage (DM basis, ▲) in a 2 × 21-day cross-over design. * Indicate an effect of diet within feeding occasion (15.00 feeding and 22.00 feeding, *p* < 0.05). Unfilled symbols differ from the prefeeding sample (T0) within that feeding occasion (*p* < 0.05).

Plasma insulin concentrations were higher on diet CON than on diet S following both meals (Figure 3, *p* < 0.01). At the 15.00 feeding, there was a significant increase after 90 and 120 min for both diets (*p* < 0.01) whereas after the 22.00 feeding, only diet CON showed an increase (Figure 3, *p* < 0.001)). There was no general difference between the diets for the plasma glucose concentrations but on diet CON there was a significant increase in plasma glucose 90 and 120 min after the 15.00 feeding (*p* < 0.01), which was not observed in diet S (Figure 3). Plasma NEFA concentration was greater on diet S than on diet CON when both feeding occasions were included in the analysis and also following the 22.00 feeding alone (*p* < 0.01). There was a significant drop in plasma NEFA with both diets following the 15.00 feeding whereas this was only observed on diet S after the 22.00 feeding (Figure 3, *p* < 0.01). In diet CON, the TPP was elevated throughout the 15.00 feeding whereas it had decreased to basal levels after 120 min on diet S (Figure 3, *p* < 0.05). At the 22.00 feeding, no elevation at all was observed in diet S. There was a significant increase in the TPP in both diets after the 15.00 feeding but only in diet CON following the 22.00 feeding. Diet and feeding occasion had no effect (*p* > 0.05) on plasma concentrations of acetate (mean 0.6 ± 0.1 mmol/L and 0.7 ± 0.1 mmol/L for CON and S respectively) or urea (8.3 ± 0.4 mmol/L and 9.0 ± 0.4 mmol/l for CON and S, respectively).

### 3.5. Feeding Time, Prefeeding Behaviour, Heart Rate, Cortisol and Serotonin Concentration

Horses were fed 50/50 of haylage 1 and haylage 2 or straw when the behaviour was studied and haylage 1 was always consumed before the straw or haylage 2 and horses spent the same amount of time consuming haylage 1 irrespective of diet (13 ± 1 min vs. 13 ± 1 min, *p* > 0.05). When fed diet S, feeding pauses were longer and total ration intake time was longer compared to when fed diet CON (Table 4). The number of oral behaviours without eating (licking lips, yawn and sniff floor) during the 15 min prior to feeding tended to be less on diet S (Table 4) but there were no differences detected between the diets in the number of motion behaviours (pawing, walking, scratching). No difference was detected between the diets regarding mean HR during the 30 min prior to feeding (C: 33 ± 1 bpm and S: 32 ± 1 bpm, *p* > 0.05).

There was no difference detected in plasma cortisol between diets during the 15.00 but during the 22.00 feeding the concentrations were higher on diet S compared with diet CON (Table 5). Plasma serotonin concentrations tended (*p* = 0.05) to be higher on diet S during the 15.00 feeding compared to when fed diet CON (Table 5).

## 4. Discussion

Based on the study findings, we suggest that inclusion of 50% straw in a grass forage diet may cause positive metabolic and behavioural changes, which could be beneficial to overweight horses, without increasing the prevalence and severity of gastric ulcers. In an earlier study, feeding with straw as the only/major roughage source was associated with a higher risk of gastric ulcers [11]. One might speculate that the slower consumption rate of straw in the present study when fed at 50% of the forage ration could be a protective factor for the development of EGUS since more frequent forage feeding (< 6 h between meals) was also shown in the previous study to decrease the likelihood of ESGD [11]. Interestingly, in the current study the prevalence and severity of gastric ulcers actually decreased over the study period. It is possible that the transportation and initial change in the environment contributed to the formation of ulcers in some of the horses, as the first gastroscopy was undertaken after the 5 day adaptation period, and the impact of these changes reduced over the study. However, the fact that there was no increase in gastric ulceration during the trial does suggest that both diets were supportive of gastric health.

One of the theoretical benefits with straw is that, if energy restriction is needed, one can keep the roughage allowance high, to support normal feeding behaviour, while still keeping energy intake low. Certainly, recent work seems to suggest that straw addition may help to promote weight loss in grazing equids [25]. As both obesity and insulin dysregulation (which includes high basal insulins as well as abnormal insulin responses to oral hydrolysable carbohydrate feeding) are risk factors for horses to develop laminitis [5,26] the results of the present study suggests that replacing half of the daily forage allowance with straw may be beneficial to horse health in individuals prone to these conditions. In the current study set up, the straw diet offered was iso-caloric with the grass forage diet (since weight loss was not aimed for) but the DM allowance was higher. However, there were more leftovers on the straw diet which resulted in unchanged actual DM intake between the diets, but a lower energy intake on the straw diet. Leftovers (0.27 kg DM/100 kg BW/day) corresponded to 19% of the allowance. This amount of leftovers is in the same range as earlier defined as ad libitum feeding [20] and implies that the straw diet was in fact being offered ad libitum but energy intake was nevertheless lower than on the grass forage diet. Ideally ad libitum forage provision should be the goal for all horses so that the horses’ natural feed seeking behaviour has a chance to be rewarded [6]. The behavioural observations also supported this in that feeding time was longer on the straw diet compared to the grass forage diet. The straw and control diets were consumed at a rate of 0.64 and 0.98 kg DM/h, respectively, which means that when on the straw diet potentially the animals could have spent 11.2 h foraging per 24 h compared to 6.2 h/24 h when on the control diet. The straw diet would thereby support the suggested minimum feeding time of 8 h/day [6], whereas the control diet would not. An 80% increase in feeding time, as shown with the straw diet, is in the same range as the increase in feeding time observed when changing from feeding on the floor to feeding from hay nets with small openings (3.2 cm, [27]). There might be several reasons for the increased feeding time on straw. Differences in palatability might be one but it is known that the higher the NDF content, the longer the feeding time [28]. However, in the current study there were no differences detected between the diets regarding NDF content. Interestingly, the horses also spent more time pausing their feed intake while on the straw diet whereas on the grass forage diet the allowance was consumed almost at once (27 vs. 1.5% of the feeding time were pauses, respectively).

The changes observed in the TPP also indicate that feeding behaviour was less voracious/rapid on the straw diet than on the grass forage diet. An increase in the TPP following feeding forage is expected since the secretion of saliva and gastrointestinal fluid causes a net loss of fluid from the extra cellular fluid to the gastrointestinal tract [29]. This effect might be more pronounced in voracious individuals [30]. Interestingly, during the evening meal there was no increase at all on the straw diet, potentially indicating a slower feed intake resulting in equilibrium between fluid secretion and absorption. If straw had been offered also with the morning meal on the straw diet (morning meals consisted of haylage only in both diets) it is possible that the TPP levels would have been stable also during the 15.00 feeding.

Since straw contains a higher proportion of indigestible fibres than grass forages, one could hypothesise that body weight would be higher on the straw diet due to more indigestible fibre (and associated water) in the gastrointestinal tract. We observed the opposite, i.e., a small decrease of 4 kg, which is most likely explained by the lower energy intake on the straw diet resulting in some loss of fat deposits. This could not be verified by the body condition scoring, but overall plasma NEFA concentration was greater on the straw diet potentially indicating the use of adipose tissue to support energy maintenance. The lack of difference in plasma urea levels indicate, however, that nitrogen balance (i.e., protein balance) was not affected on the straw diet. This is however expected since the digestible CP requirement was met [31] and at the same level in both diets.

Generally, a drop in NEFA can be expected after feeding in meal fed horses [32,33] but this did not happen in the evening on the grass forage diet. This indicates that the horses were still in a fed state prior to the evening meal on this diet and that NEFA levels therefore were not altered much. This is also in accordance with the feeding regimen, where the shortest feeding interval was between the afternoon and evening meal and the energy allowance in the afternoon was of the same size as the evening meal (35% of the total daily allowance).

The plasma insulin response was lower on the straw diet than on the grass forage diet, which could be expected based on their WSC contents. Interestingly, during the evening meal there was no significant increase at all in insulin when on the straw diet, again possibly reflecting the slower feed intake behaviour. It is also possible that plasma glucose levels at this time needed to be supported by glucogenolysis, which could explain the elevated cortisol levels in the evening on the straw diet (since cortisol helps maintain blood glucose levels). No changes in plasma glucose concentrations were observed on the straw diet whereas a significant increase was observed on the grass forage diet following the afternoon feeding. Although the WSC content was lower on the straw diet this cannot be generalised to all wheat straw batches. The chemical composition may vary according to agronomic conditions, location, climate in addition to heritable variation in different wheat lines [34]. Tishler*,* et al. [35] screened 48 samples of wheat straw and found the major sugar monomers being fructose and glucose. Most samples had free sugars less than 30 g/kg straw but samples with 130 g sugar/kg were also found.

One could speculate on other reasons for the higher plasma cortisol concentration on the straw diet during the evening feeding. Higher physical activity or stress/excitement could be options. However, we did not detect any differences in motion behaviours between diets and there were also no differences in heart rate in relation to feeding, so physical activity/stress was probably not of importance for the cortisol elevation. In contrast, the number of oral behaviours prior to feeding tended to be higher on the grass forage diet than on the straw diet, which might indicate differences in the motivation for feed seeking and possibly higher motivation on the grass forage diet.

Interestingly, in the afternoon the serotonin levels tended to be higher on the straw diet compared to the grass forage diet. Higher plasma serotonin levels have earlier been observed in horses fed a high fibre diet compared to horses fed a high starch diet [36] although the authors provide no mechanistic explanation for this. The observations made in both studies could theoretically be due to differences in the tryptophan intake (the precursor of serotonin) but we suggest that it is linked to satiation. The function of serotonin is complex and until recently, most studies have focused on its effects on the central nervous system, mood, behaviour, and anxiety [37]. Low serotonin levels have been linked to low mood states. Chewing appears to activate brain serotonergic neurons and activation of central serotonin appears to suppress feeding [38]. Peripherally administered 5-HT also decreases food intake in rats [39]. In humans, prolonged mastication reduces self-reported hunger levels [40]. If all this is true also for horses, satiation might be one plausible explanation for the lower feed intake on the straw diet and for horses taking more ingestion breaks/pauses on this diet.

The majority of the body’s serotonin is found in the gut [41] in serotonin synthesizing neurons and in mucosal cells of the epithelium [38]. Serotonin synthesizing neurons supports intestinal motility [38]. It has been suggested that gut-derived serotonin plays a crucial role in adipose tissue metabolism [37]. Peripheral serotonin increases circulating free fatty acids by stimulating lipolysis and is important in fasting adaptation [42]. This mechanism is in accordance with observations in the present study, i.e., elevated plasma NEFA levels on the straw diet. The effect of serotonin on the gut motility of horses is not well understood as far as we know. In the present study, gut activity (sounds) appeared to reflect normal motility patterns, i.e., almost continuous borborygmi [43] on both diets, and there were no differences detected between diets. Faecal water was also within normal ranges and did not differ between diets. These observations, along with no other health disturbances, indicate that no major adverse effects on gut function were imposed by either of the diets. One could speculate that the study set up itself would have been a challenge to gastrointestinal health (three feed changes in 7 weeks). Feed changes have been associated with increased risk of colic during the following subsequent 2 weeks [44]. However, the same study setup (abrupt feed changes or changes within 4 days) have been used in many studies with forage-only diets without problems being encountered [21,45,46,47,48,49,50,51,52]. This is in contrast to the field observations by Hudson et al. [44] and may be due to that our feed changes have been between feeds of good hygienic quality and “balanced” (as in the present study), i.e., diets have had similar energy and nutrient contents, with a few exceptions where crude protein content differed substantially [45,47,48,50,52]. However, certain individuals and maybe breeds may be more prone to impactions. As earlier suggested by Harris et al. [6], we advise that any feed and forage changes are made gradually over several days and possibly several weeks especially if the diets differ significantly in energy, carbohydrate and crude protein content.

## 5. Conclusions

Although this was a small short-term study it suggests that wheat straw of good hygienic quality can be included at up to 50% of the diet of forage fed horses without any apparent increased risk of gastric ulcers. In addition, it slows feed intake and extends the time spent foraging beyond that expected from the dry matter provision. Straw seems also to promote satiety and a metabolic profile suitable for overweight horses and may thereby improve welfare for horses with low energy requirements. Straw may accordingly also be an effective component of a weight loss management programme. However, as the present study was performed using a low number of overweight horses, further studies are required to confirm this.

## Figures and Tables

**Table 1:** Dry matter, energy and nutrient content per kg dry matter (DM) in the feeds used in the study.

Item	Grass Haylage 1	Grass Haylage 2	Wheat Straw
Dry matter (DM), %	59	49	89
Metabolisable energy (ME), MJ	11.2	9.8	6.7
Crude protein (CP), g	180	79	22
Digestible crude protein (dCP), g	137	43	9
Neutral detergent fibre (NDF), g	483	608	802
Water soluble carbohydrates (WSC), g	77	49	8
Ca, g	3.9	6.2	2.0
P, g	2.6	1.7	0.3
Mg, g	1.7	1.2	0.5

**Table 2:** Dry matter, energy and nutrient allowances ^1^ of the control diet (CON, (grass haylage only)) and the straw diet (S, 50% grass haylage and 50% wheat straw on a dry matter basis) per 100 kg BW and day (LSmeans ± SE) offered to six horses in a 2 × 21-day cross-over design. The diets were fed on metabolic body weight basis and allowances rounded to the nearest 10 g.

Item	Control Diet ^1^	Straw Diet ^1^	*p*
Dry matter (DM), kg	1.22 ± 0.01	1.43 ± 0.01	<0.05
Metabolisable energy (ME), MJ	12.9 ± 0.6	12.7 ± 0.6	>0.05
Crude protein (CP), g	158 ± 7	141 ± 7	>0.05
Digestible crude protein (dCP), g	111 ± 5	103 ± 5	>0.05
Neutral detergent fibre (NDF), g	667 ± 36	938 ± 36	<0.05
Water soluble carbohydrates (WSC), g	77 ± 3	60 ± 3	<0.05
Ca, g	7.1 ± 0.3	5.2 ± 0.3	<0.05
P, g	3.6 ± 0.2	3.1 ± 0.2	<0.05
Mg, g	2.8 ± 0.1	2.7 ± 0.1	>0.05
Na, g	3.9 ± 0.2	3.9 ± 0.2	>0.5

^1^ Contribution of DM, ME, CP, NDF and WSC from the mineral and vitamin balancer is not included. The balancer provided additional vitamins and trace elements/100 kg BW: Cu 16 ± 2 mg, Se 0.3 ± 0.0 mg, I 0.05 ± 0.01 mg, Fe 10 ± 1 mg, Mn 17 ± 2 mg, Co 0.17 ± 0.02 mg, Zn 43 ± 5 mg, vitamin A 1730 ± 204 IU, vitamin D3 173 ± 20 IU, vitamin E 87 ± 10 IU.

**Table 3:** Intake of dry matter (DM), metabolisable energy (ME), crude protein (CP), digestible crude protein (dCP), neutral detergent fibre (NDF) and water soluble carbohydrates (WSC) per 100 kg body weight and day (LSmeans ± SE) in six horses fed a diet with 50% wheat straw and 50% grass haylage (DM basis) and a control diet without straw (grass haylage only) in a 2 × 21-day cross-over design.

Item	Control Diet	Straw Diet	*p*
DM, kg	1.2 ± 0.06	1.2 ± 0.06	>0.05
ME, MJ	12.4 ± 0.5	10.7 ± 0.5	<0.001
CP, g	155 ± 5	134 ± 5	<0.0001
dCP, g	108 ± 3	99 ± 3	<0.0001
NDF, g	633 ±41	696 ± 41	>0.05
WSC, g	75 ± 2	60 ± 2	<0.0001

**Table 4:** Behaviours observed prior (15 min) and during feeding (LSmeans ± SE) in six horses ^1^ fed 50% (DM basis) haylage 1 and either 50% haylage 2 (control diet) or 50% straw (straw diet) in a 2 × 21-day cross-over design. During one hour after the feed was offered, number of minutes where feed was available (possible feeding time (including feeding pauses), and minutes of feeding pause (no feed intake but feed still available) was registered and after 60 min feed leftovers were weighed.

Item	Control Diet	Straw Diet	*p*
Oral behaviours prior to feeding	11 ± 1	6 ± 2	0.07
Motion-related behaviours prior to feeding	6 ± 1	5 ± 2	>0.05
Feeding pause, min	1 ± 2	16 ± 2	<0.001
Possible feeding time, min	44 ± 3	54 ± 3	<0.05
Feed left overs, kg	0.1 ± 0.0	0.2 ± 0.0	<0.05

^1^ Horses with body weight 450–550 kg and 200–250 kg were fed 1 kg and 0.5 kg DM, respectively.

**Table 5:** Plasma cortisol and serotonin concentrations (LSmeans ± SE) during feeding at 15.00 and at 22.00 in six horses fed a control diet of grass haylage only and a diet with 50% wheat straw and 50% grass haylage (of DM) in a 2 × 21-day cross-over design. Results shown as a mean from blood samples collected before, 90 and 120 min after feeding.

Item	Control Diet	Straw Diet	*p*
Cortisol feeding 15.00, nmol/L	210 ± 60	210 ± 60	>0.05
Cortisol feeding 22.00, nmol/L	130 ± 60	176 ± 60	<0.05
*P* feeding occasion	<0.001	0.09	
Serotonin feeding 15.00, nmol/L	162 ± 55	261 ± 55	0.05
Serotonin feeding 22.00, nmol/L	211 ± 55	218 ± 55	>0.05
*P* feeding occasion	>0.05	>0.05	

## Data Availability

The data presented in this study are available on request from the corresponding author.
